# Socioeconomic status, health inequalities and non-communicable diseases: a systematic review

**DOI:** 10.1007/s10389-017-0850-z

**Published:** 2017-10-17

**Authors:** Santiago Lago, David Cantarero, Berta Rivera, Marta Pascual, Carla Blázquez-Fernández, Bruno Casal, Francisco Reyes

**Affiliations:** 1 GEN Governance and Economics Network-Spain, Faculty of Business and Tourism University of Vigo, Campus Universitario As Lagoas s/n, 32004 Ourense, Spain; 2 Department of Applied Economics, Faculty of Business and Tourism University of Vigo, Campus Universitario As Lagoas s/n, 32004 Ourense, Spain; 3 Department of Economics, Faculty of Business and Economics University of Cantabria, Avda. de los Castros, S/N, 39005 Santander, Spain; 4 Department of Applied Economics, Faculty of Business and Economics University of A Coruña, Campus de Elviña, 15071 A Coruña, Spain

**Keywords:** Socioeconomic status, Health inequalities, Non-communicable diseases, Systematic review

## Abstract

**Aim:**

A comprehensive approach to health highlights its close relationship with the social and economic conditions, physical environment and individual lifestyles. However, this relationship is not exempt from methodological problems that may bias the establishment of direct effects between the variables studied. Thus, further research is necessary to investigate the role of socioeconomic variables, their composition and distribution according to health status, particularly on non-communicable diseases.

**Subjects and methods:**

To shed light on this field, here a systematic review is performed using PubMed, the Cochrane Library and Web of Science. A 7-year retrospective horizon was considered until 21 July 2017.

**Results:**

Twenty-six papers were obtained from the database search. Additionally, results from “hand searching” were also included, where a wider horizon was considered. Five of the 26 studies analyzed used aggregated data compared to 21 using individual data. Eleven considered income as a study variable, while 17 analyzed the effect of income inequality on health status (2 of the studies considered both the absolute level and distribution of income). The most used indicator of inequality in the literature was the Gini index.

**Conclusion:**

Although different types of analysis produce very different results concerning the role of health determinants, the general conclusion is that income distribution is related to health where it represents a measure of the differences in social class in the society. The effect of income inequality is to increase the gap between social classes or to widen differences in status.

## Introduction

From a broad point of view, an individual’s health is considered not only an absence of disease, but also a fundamental human right (WHO 1986). A comprehensive approach to health highlights its close relationship with social and economic conditions, the physical environment and individual lifestyles. According to the Commission on Social Determinants of Health, we can consider health inequalities to be the result of the cumulative impact of decades of exposure to health risks of those who live in socioeconomically less advantaged circumstances (WHO 2008).

If we focus on all the socioeconomic variables, the relationship between income (understood as a measure of socioeconomic status) and health is probably the most complicated (Fuchs 2004). The correlation coefficient, obtained from the crudest associations, can range from highly positive to slightly negative, depending on the context and the aggregation level. Even when the positive correlation is strong and stable, causal interpretations may include income influencing health, health influencing income and/or “third variables” affecting both indicators in the same direction and at the same time. For this reason, the gross domestic product (GDP) is related to some health outcomes indicators (Kanavos and Mossialos 1996). However, there are exceptions. For example, some Southern countries in the European Union that are relatively poor have a life expectancy indicator greater than that of the rich countries of Northern Europe. Also, we can observe that the USA, one of the world’s richest countries in terms of GDP per capita, has infant mortality rates similar to those of poorer countries (Starfield 2000).

In addition, there is a large and growing body of literature in which the effects of income on health are examined because of the importance of these effects in the development of appropriate economic policies (Gravelle et al. 2002). Many studies have shown a negative association between income and mortality (Lutter and Morrall 1994; McCarron et al. 1994; Viscusi 1994; Singh and Siahpush 2002; Shaw et al. 2005; Pearce and Dorling 2006; Leyland et al. 2007; Ezzati et al. 2008; Thomas et al. 2010). These empirical findings suggest that individual health is a function of individual income—*the absolute income hypothesis*. In relation to income inequality, the *relative income-health hypothesis* suggests that income inequality has a detrimental effect on population health because it is an individual’s relative rather than absolute income that is important for health (Marmot et al. 1991; Wilkinson 1997, 1998; Wildman 2001, 2003; López I Casasnovas and Rivera 2002; Gravelle et al. 2002; Eberstadt and Satel 2004). Income inequality may therefore be a health risk (Le Grand 1987; Wilkinson 1992, 1996). Thus, life expectancy and population mortality have been used as key indicators of economic and social development (Van Doorslaer and Koolman 2004; Cantarero et al. 2005).

Although previous empirical literature presents different interpretations of the evidence, most analyses report that the average health is worse in more unequal societies. However, this relationship is not perfect, since there are several determinants that can affect it. There is clear evidence indicating that a nonlinear, typically concave relationship between health and income at an individual level will generate an aggregate relationship in which average health will depend negatively on the degree of inequality in the income distribution (Duleep 1995; Wilkinson 1996; Mackenbach et al. 2005; Mackenbach 2012). Hence, income redistribution from the rich to disadvantaged groups may improve some health indicators (Kawachi and Kennedy 1999). Also, some authors have suggested the existence of conceptual difficulties in studying the relationship between income and individual health when aggregated data are used, because revenues have a diminishing marginal effect on health (Deaton and Muellbauer 1980). This is because if income inequality increases, it tends to reduce average health but improve the health of “the rich,” although this latter effect is less significant than the reduction in overall health.

## Methods

A systematic literature search was performed in PubMed, the Cochrane Library and Web of Science (until 21 July 2017) to identify the most relevant published evidence regarding the relationship between income and health. In all databases, terms related to “health,” “income” and “inequalities” were combined. See Appendix Table 4 for the search strategy/search terms used. The searches were confined to papers published in the English language since 2010 to limit the scope of this review to the most recent data and the state of the art. In other words, we considered this retrospective horizon, from the beginning of this century up to date, to be enough.

## Results

**Fig. 1:**
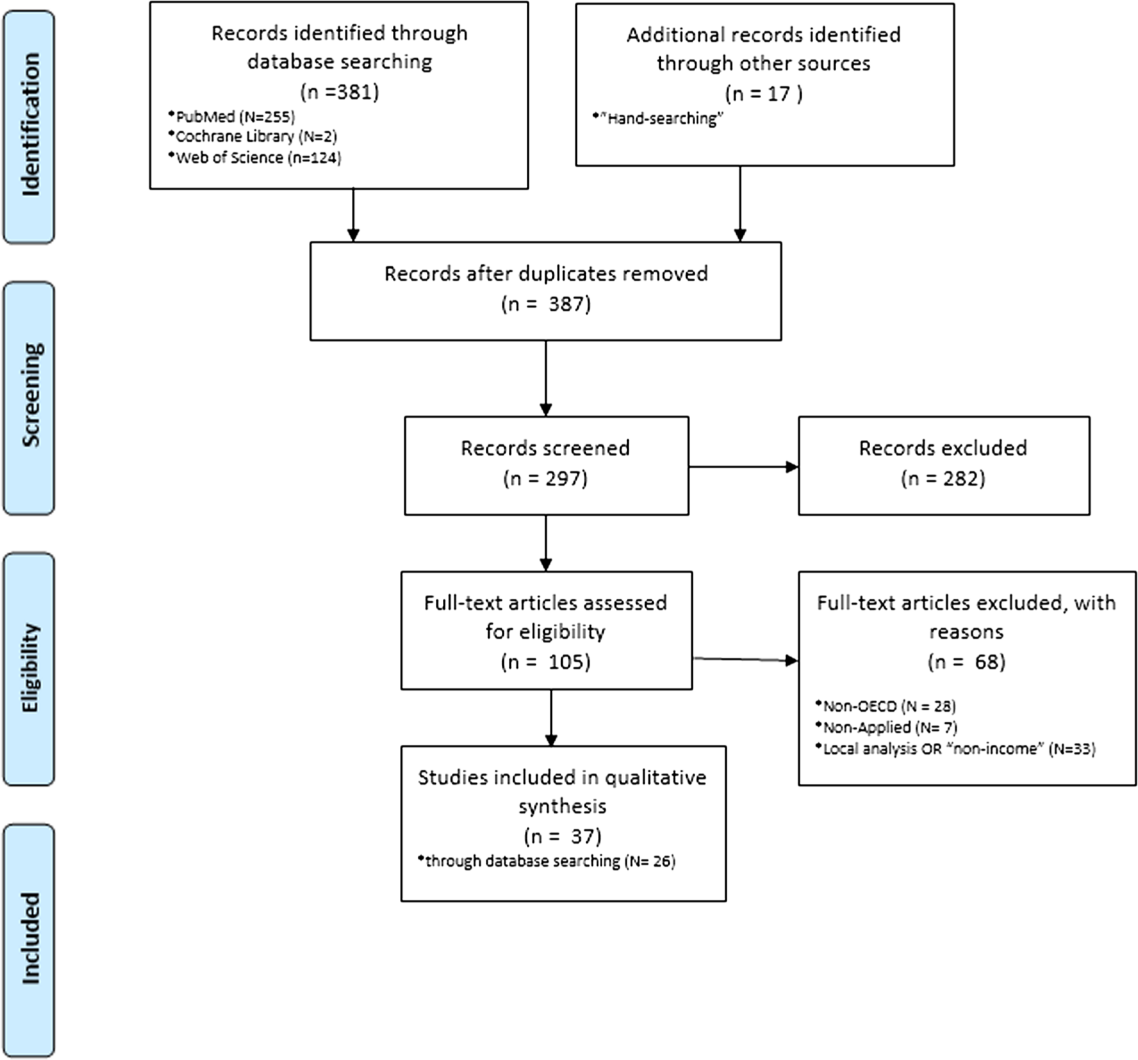
Flow diagram of the paper selection process. The flow diagram depicts the different phases (identification-screening-eligibility-included) of the systematic review. It maps out the number of records in each phase and shows how many studies were included or excluded, respectively

After finding publications in the electronic searches, duplicate records were removed. The selection of papers was ultimately based on the following eligibility criterion: an applied study with a focus on one or more OECD countries (including the European Union and other developed countries). Additionally, the results of “hand searching” were also included in the following pages, where a wider horizon was considered. Figure 1 is a diagram of the paper selection process.

The literature search located 381 publications in the databases under consideration, and 17 papers published between 2010 and 2017 were identified through “hand searching.” A total of 11 duplicates were removed, resulting in 387 “unique papers.” After screening the titles against the eligibility criteria, 105 papers were selected. Of these, 68 articles were excluded as they did not fit the previous criteria. So, a final set of 37 selected studies were taken into account in this review, and further papers were finally considered to have a robust overview. Table 1 focuses on the 26 papers found in the database search.

**Table 1:** Characteristics of the studies included in the review (omitting those found by “hand searching”)

Study	Year	Country	Methodology	Main result
Allanson et al. (2010)	1991–1999	England	Index of “income-related health mobility”	There has been a fall in income-related health inequality
Elgar (2010)	2005–2008	33 Countries	2-Level linear model with variances	Income inequality might contribute to short life expectancy and adult mortality in part because of societal differences in trust. [Income inequality correlated with country differences in trust (*r* = −0.51), health expenditures (*r* = −0.45), life expectancy (*r* = −0.74) and mortality (*r* = 0.55). Trust correlated with life expectancy (*r* = 0.48) and mortality (*r* = −0.47) and partly mediated their relations to income inequality. Health expenditures did not correlate with life expectancy and mortality, and health expenditures did not mediate links between inequality and health]
Huijts et al. (2010)	2002, 2004, 2006	Denmark, Finland, Norway and Sweden	Binomial logistic regression models	Income gradient. People reported significantly better health and were less likely to suffer from long-term illnesses if they had a higher income
Idrovo et al. (2010)	2002–2004	110 Countries	Path analysis of cross-sectional ecological data	Income inequality and social capital have direct effects on life expectancy at birth. [The correlations between the variables studied were statistically significant (*p* < 0.05) and displayed the desired tendency. Where LEB was lower, income inequality, political rights and civil liberties or ethnic fractionalization were greater. In addition, LEB was higher when less corruption was perceived and where there is more generalized trust. The Gini coeffi cient was greater where the ethnic fractionalization was greater and lower where there was less perceived corruption. The Gini coeffi cient was negatively correlated with generalized trust and the CPI 2004, and it was positively correlated with ethnic fractionalization and the index of freedom. The correlations between the CPI and the index of freedom or ethnic fractionalization were negative, indicating that fractionalization and political rights and civil liberties were greater where less corruption was perceived. Ethnic fractionalization was positively correlated with the index of freedom and negatively correlated with generalized confidence. Finally, the index of freedom was negatively correlated with generalized trust]
Islam et al. (2010)	1980–1981, 1988–1989, 1996–1997	Sweden	Concentration index, by fixed effect model	Conventional unstandardized and standardized (by age and gender) CIs (concentration indexes) increase over time. [Contribution of income to health inequality decreases over time (from about 19% in wave 1 to 9% in wave 3), which suggests population aging weakens the relative importance of income on total health inequality]
Karlsson et al. (2010)	2006	21 Countries	Ordered probit model	There is evidence of a negative relationship between income inequality and individual health in high-income countries
Oshio and Kobayashi (2010)	2001, 2004, 2009; 2000, 2003, 2006	Japan	ANOVA and ordered bivariate probit models	Individuals who live in areas of high inequality tend to report themselves as both unhappy and unhealthy
Petrie et al. (2011)	1999–2004	Scotland, England and Wales	Decomposition method to account explicitly for mortality in the longitudinal analysis of income-related health inequalities	Accounting for deaths in the decomposition analysis shows that the relative health changes for both regions and genders between 1999 and 2004 were significantly regressive, such that initially poor people experienced a greater share of health losses compared to their initial state of health. [Income-related health mobility, expressed as a proportion of final health inequality, was 11.5% for *CI* ^u^ and 15.9% for *CI*, with the latter value being 35.3% larger than the former where this ratio is also given by the ratio of the corresponding *P* values]
Chen and Crawford (2012)	2000	US	Multilevel regression models	Income inequalities measured at different geographic scales have different interpretations and relate to societal factors at different levels. A rejection of the IIH (income inequality hypothesis) at one geographic level cannot negate positive evidence at another level
Hosseinpoor et al. (2012)	2002–2004	48 Countries	Bivariate and count data models	Prevalence of non-communicable disease risk factors demonstrates different patterns for varying degrees of socioeconomic inequality across low- and middle-income settings. [In both sexes, current daily smoking and low fruit and vegetable consumption were more prevalent in the poorest wealth quintile than in the richest, and regular inequality in both absolute and relative terms was found after controlling for respondents’ age and country of residence (model 1). The highest absolute inequality across the entire distribution of wealth was related to smoking among men living in LICs (prevalence difference: 23.0%, 95% CI: 19.6, 26.4). The absolute difference of low fruit and vegetable consumption prevalence between poorest and richest adults of the study LIC group was near 10% (males: 9.7%, 95% CI: 6.2, 13.2; females: 9.5%, 95% CI: 6.5, 12.4). SII values for smoking and low fruit and vegetable consumption were not significantly different in LIC and MIC study groups, except smoking in men, which demonstrated significantly higher absolute inequality in the LIC group. On the contrary, physical inactivity illustrated in reverse inequality, with elevated prevalence in populations of high socioeconomic status. Reverse inequality was pronounced in the LIC group. Inadequate physical activity among the poorest adults in LICs was about half as prevalent as among the richest (prevalence ratio: males: 0.46, 95% CI: 0.33, 0.64; females: 0.52, 95% CI: 0.42, 0.65. Heavy episodic alcohol drinking demonstrated mixed patterns of inequality. For instance, women in LIC and MIC groups showed regular and reverse inequality, respectively (prevalence ratio: LICs: 2.51, 95% CI: 1.68, 3.74; MICs: 0.65, 95% CI: 0.47, 0.90), although the absolute inequality in both groups was very low (about 1 percentage point). No inequality was reported by men in the MIC group, and regular inequality was weakly demonstrated by men in the LIC group (prevalence ratio: 1.41, 95% CI: 1.03, 1.93)]
Karlsdotter et al. (2012)	2007	Spain	Logit model	Support for the absolute income hypothesis: “a higher level of personal income is correlated with a lower probability of negative health outcomes”
Martinson (2012)	1999–2006; 2003–2006	US and England	Weighted prevalence rates and risk ratios by income level for different health risk factors or conditions (obesity, hypertension, diabetes, low high-density lipoprotein cholesterol, high cholesterol ratio, heart attack or angina, stroke, and asthma)	Income gradients in health are very similar across age, gender and numerous health conditions and are robust to adjustments for race/ethnicity, health behaviors, body mass index and health insurance
Allanson and Petrie (2013)	1999–2004	Great Britain	Dynamic health function modeling framework (two-part model). Changes in IRHI (income related health inequality) through both morbidity changes and mortality. Quality adjusted life years (QALYs) as health measure	Major driver of the no equalizing effects of mortality is the positive association between age and poverty, with other significant contributors including initial health status, advanced levels of educational attainment, gender and smoking
Ásgeirsdóttir and Ragnarsdóttir (2014)	2007, 2009	Iceland	Health concentration index	Cyclical income-related health distributions. [For males education also contributed somewhat to inequality, or around 5% for high education in 2007 and low education in 2009. The largest increase in contributions to male inequality between years is from being a student, with a 22.96 percentage point increase in contributions when individual income was the income measure, 7.08 percentage points when household income was the income measure and 8.28 percentage points when equalized household income was the income measure. For females being a student reduced inequality between years, but mainly when individual income was the income measure, with a 5.91 percentage point change. For males the contribution of being single increased by 4.18 percentage points between years using individual income \as the income measure. For males the contribution of being retired decreased by around 5–6 percentage points between years as well as the contribution of being \unemployed, which decreased substantially using all income measures. The largest decrease was when individual income was used, or 20.69 percentage points. The contributions of being a male business owner increased by 4.38 percentage points between years when individual income was the income measure and being disabled by 5.76 percentage points. The contribution of being a male former smoker increased by 4.75 percentage points between years when individual income was the income measure while the contribution of smoking daily increased by 2–3 percentage points between years for females, using all income measures. For males the contribution of age increased by 10.99 percentage points when household income was the income measure and 5.90 percentage points when equalized household income was the income measure, while it decreased slightly between years for females. The change in contribution of equalized household income to health inequality for males between years was 19.37 percentage points. For females the contribution of individual income reduced by 7.26 percentage points between years]
Siegel et al. (2015)	2002, 2006	Germany	Semiparametric extension of Wagstaff’s corrected concentration index	The degree of deprivation-specific income-related inequality in the three health outcomes exhibits only small variations across different levels of multiple deprivation for both sexes. [Health inequalities with respect to household income are considerably stronger than those with respect to small area deprivation. All differences are highly significant (*p* < 0.01) in the female sample. In the male sample, the differences are statistically significant at the 99% level for income and obesity and at the 95% level for diabetes. No income-related gradient in the distribution of obesity is observed within the most deprived 10% of the communes among males, income-related inequalities of hypertension among males hardly exist in the worst-off 50% of communes, and the income-related inequality of diabetes among males varies around ≈ −0.1]
Siegel et al. (2014)	1994–2011	Germany	Health concentration index	Income-related health inequalities have roughly doubled over time, to the disadvantage of the economically deprived
Vallejo-Torres et al. (2014)	2006–2010	England	Health concentration index	Inequalities occur across the life course, but for some health issues there may be a period of equalization in late adolescence and early adulthood. [The income effect among children and young adolescents was not significantly different, but the results showed in some cases a different impact between those aged 12–15 years and those aged 16–19. The impact of income on health among late adolescents (aged 16–19 years) and young adults (aged 20–24 years) was not significantly different, but the impact in mid-adults (25–44 years) was significantly different from that in young adults in some instances, especially among females. Among males the income effect was not statistically significantly different until comparing 25–44-year and 45–64-year age groups for most indicators. For both genders and in almost every health indicator, the impact of income was significantly different in the elderly compared with older adults]
Torre and Myrskylä (2014)	1975–2006	21 Developed countries	Time series	Income inequality is positively associated with mortality of males and females between the ages of 1 and 14 years and 15 and 49 years, and with mortality of females between the ages of 65 and 89, albeit less strongly than for younger age groups. [The coefficient 0.055 for men suggests that a 1% increase in GDP per head leads to a 0.055% increase in life expectancy. Among women, the coefficients are positive for all age intervals but significant at *p* = 0.05 level only up to age 15 and with *p* = 0.10 level up to age 50. For older ages, the coefficients are statistically insignificant. The coefficient 0.47 for infant mortality (both males and females) suggests that a 1% increase in income inequality would increase infant mortality by 0.47%. For an increase in the Gini index, a 43% increase in income inequality would correspond to 14% (1.43^0.373) increase in child mortality for men and 16% (1.43^0.424) increase for women. However, since the mortality rate at ages 1–14 is lower for women than for men (in our data, on average 25% lower), the absolute effect is greater for men. At ages 15–49, the male mortality coefficient for the Gini index is still positive (0.285) and highly significant (*p* < 0.10) However, for women aged 15–49 the coefficient size is 40% smaller than for men (0.171) and significant only at *p* < 0.10. For the GDP per head predictor, for both men and women, the coefficient -0.30 at ages 1–14 suggests that a doubling of GDP per head would decrease child mortality by 30%]
Chauvel and Leist (2015)	2005, 2011	18 Countries	Multilevel models	Linear health gradient increase. Intergenerational transmission of status gains in importance in countries with higher income inequality. [The stratification variables were moderately but not overly correlated (Pearson’s correlation of logit rank transformed social origins and income *r*_*P*_ = 0.20, *p* < 0.001; Spearman’s correlation of education and occupation *r*_*S*_ = −0.54, *p* > 0.001; Spearman’s correlations of education and occupation, respectively, with logit rank transformed social origins and income −0.37 < *r*_*S*_ < 0.36, *p* < 0.001)]
Jutz (2015)	2008–2009	42 European countries	Two-step hierarchical estimation approach.	Income inequality has more impact on health inequalities than do social policies. [The relation between social protection expenditures and health inequalities could not be confirmed. As expected, SPE and health inequalities are negatively related (−0.25), but the relation does not reach significance. The Gini index was positively related to health inequalities (0.39, *p* < 0.05), i.e., higher income inequality was linked to higher health inequalities]
Lillard et al. (2015)	1913–2009, 1984–2009	US	Ordered probit models	Exposure to income inequality in early life is related to worse health in later life
Quon and McGrath (2015)	1994–1995	Canada	Multilevel modeling	Income inequality is associated with injuries, general physical symptoms, and limiting conditions, but not associated with most adolescent health outcomes/behaviors. Income inequality has a moderating effect on family socioeconomic status for limiting conditions
Rambotti (2015)	1999	US (plus international comparisons)	Bivariate and cross-sectional associations.	Poverty has a significant and adverse effect
Kim (2016)	1980s and 1990s	US	Linear probability models	Linkages were identified between state-level spending on welfare and education and lower individual risks of dying, particularly from coronary heart diseases.
López et al. (2016)	1998–2011	US	Multivariable linear and Poisson regressions	Income inequality is independently associated with higher health care expenditures and more health care use
Wilson et al. (2017)	2000–2011	US	Logistic regressions.	Racial disparities in health outcomes exist. Race/ethnic disparities are not merely a result of income

We also reviewed the works obtained from “hand searching,” where the results are almost all based on economic criteria. Specifically, we can highlight that there is also much evidence for the effect of income on health status for different socioeconomic groups. References are listed at the end of this article, and we included references in journals and cited books.

**Fig. 2:**
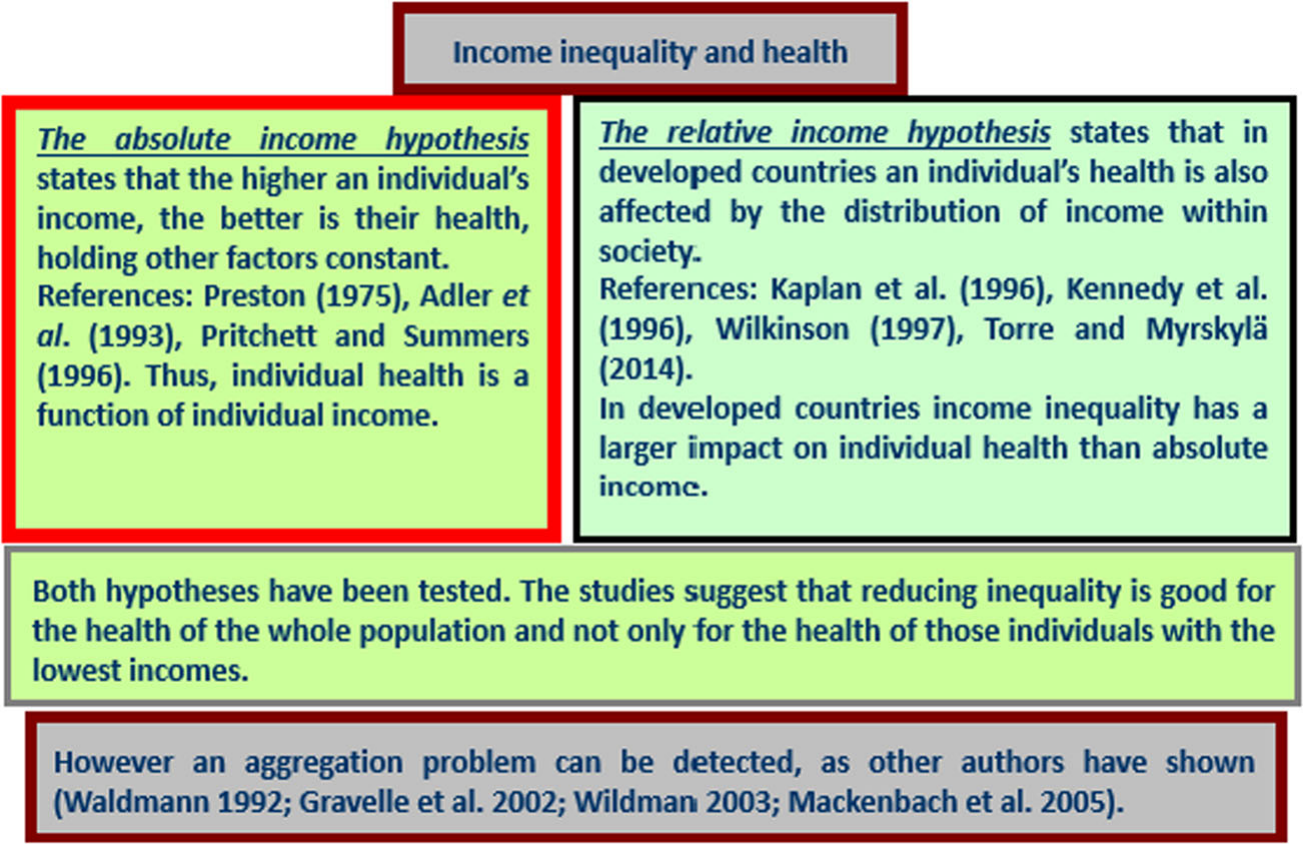
Relative and absolute income-health hypotheses

Table 1 focuses on the 26 papers obtained from the database search. The first group of studies explores the fact that people who live in areas of high inequalities tend to report themselves as having both an objective (having a shorter life expectancy and high adult mortality) and subjective health status and that this tendency increases over time (Allanson et al. 2010; Elgar 2010; Huijts et al. 2010; Idrovo et al. 2010; Islam et al. 2010; Karlsson et al. 2010; Oshio and Kobayashi 2010; Petrie et al. 2011). Moreover, the following group of studies developed various econometric approaches (multilevel regression, bivariate and count data or logit models) to consider geographic, socioeconomic and poverty-related issues (Chen and Crawford 2012; Hosseinpoor et al. 2012; Karlsdotter et al. 2012; Martinson 2012; Allanson and Petrie 2013; Ásgeirsdóttir and Ragnarsdóttir 2014; Wilson et al. 2017). The health concentration index and its corrections have been employed in recent studies (Siegel et al. 2014; Vallejo-Torres et al. 2014; Siegel et al. 2015). As described in Table 1 and Fig. 2, the relative income-health hypothesis was also analyzed by Hu et al. (2015), who conclude that in European countries income inequality does not have an independent effect on mortality.

Moreover, it seems logical that there would be a difference between rich and poor countries in how income distribution would affect health status (Waldmann 1992; Rodgers 1979; Deaton 2001b). Wilkinson and Pickett (2006) performed a review of the literature analyzing the association between the income distribution and health of the population. The general conclusion is that income distribution is related to health where it represents a measure of the differences in social class in the society.

Among the studies conducting their analysis on individual data, Ettner (1996) estimates the effects of income on a set of individual health proxies. The results show a strong positive effect of income on health. Dahl et al. (2006) analyzed the degree to which contextual income inequality affects the health in Norwegian regions; the results differ from previous studies in suggesting that in Norway a comparatively egalitarian income distribution interferes with the emergence of regional-level income inequality effects on mortality. From another point of view, a study developed in Spain (Regidor et al. 2014) showed that inequality in the distribution of provincial income declined during the 4 decades covered by the study. More recently, Pickett and Wilkinson (2015) conducted a new review of the literature on the subject, exploring the causal role of wider income differences in health. The authors highlight that the effect of income inequality is to increase the gap between social classes or to widen differences in socioeconomic status.

As Table 2 shows in relation to *health indicators*, measuring the health status of a population is problematic because there is no complete and comparable health index among countries or regions.

**Table 2:** Health indicators, data aggregation and causality of variables (income and health)

Health indicators	There is no complete and comparable health index for all countries. The indicators commonly used are mortality rates (infant and adult) and life expectancy. However, these indicators are not sensitive to improvements in quality of life (Parkin et al. 1987). Data at the individual level are recommended (Wagstaff and Van Doorslaer 2000)
Data aggregation	This presents problems from a methodological point of view. The availability of comparable data for long periods of time is a problem, and individual conditions of linearity are required, while the evidence suggests that relationships in this regard are configured in a nonlinear way (Preston 1975; Rodgers 1979; Duleep 1995; Ettner 1996; Deaton 2001a, 2001b; Gravelle et al. 2002; Mackenbach et al. 2005)
Causality of the variables	Population health would also help explain differences in income levels among individuals and countries. The effect could bias results and make any inferences about the structural effect of income on health difficult (Fuchs 1974, 2004; Ettner 1996)

*Data aggregation*, used in numerous studies examining the health status of the population in different countries and its relationship to the level of income, can also present problems from a methodological point of view. The first problem is the availability of comparable data for long periods of time. The observations are often measures at the national or regional level, in contrast to individual panel data for which there are many observations of cross-sectional measurements at very few points in time. Therefore, the problems differ depending on the observation unit adopted: the individual or an aggregated geographical area.

*Causality of the variables* that are considered in the analysis of the relationship between income and health is another methodological aspect that is particularly relevant (Fuchs 2004). Although numerous studies indicate a positive relationship between health and income, few of them analyze the causality of this association. The stability of income inequality over time in most countries makes this causality difficult to test (Babones 2008). This author points out that although there exist a “strong, consistent and statistically significant correlation between national income inequality and population health,” there is also evidence indicating that this correlation is causal.

McKeown (2009) describes changing patterns of population age distributions, mortality, fertility, life expectancy and causes of death. However, within countries, differences in living standards establish a social order in the population. Among the papers that raise the issue of income distribution as a possible reason for inequalities in individuals’ health, that published by Deaton (1999) is notable. Furthermore, Deaton and Paxson (2001) develop a similar analysis to examine the relationship between income inequality and mortality. The results show that neither the trends in the level of income nor the inequalities in income explain the adjusted mortality rates by age. Besides, Wagstaff and Van Doorslaer (2000) review a large body of literature on the effects of income inequality on population health. The literature review shows that the individual level studies considered to be relevant provide strong support for the absolute income hypothesis, no support for the relative income hypothesis and little or no support for the income inequality hypothesis. In relation to this, we can think about the countries of Eastern Europe, where, despite their egalitarian distribution of income, there are high mortality rates. Contoyannis and Foster (1999) found that it is absolute income that has a significant effect on health, not relative income. Along the same lines, we can point to the paper of Van Doorslaer et al. (1997), whose results support the idea that health inequalities cannot be definitively attributed to income inequalities.

Also, as Table 3 describes, the trajectories of social mobility over the life course (U-shaped) and the variations in patterns of social mobility mean that it is very important to study inequalities in health and socioeconomic status because they are present early in life (Currie and Madrian 1999; Bengtsson and Mineau 2009; Almond and Currie 2011; Currie and Almond 2011; De Ree and Alessie 2011; Lundborg et al. 2014).

**Table 3:** Social mobility over the life course: some findings

Recent papers	Currie and Madrian (1999), Bengtsson and Mineau (2009), Almond and Currie (2011), De Ree and Alessie (2011), Lundborg et al. (2014), Flores et al. (2015)
Some empirical findings	Inequalities in health and socioeconomic status are present early in life Childhood circumstances have direct and indirect impacts (through mediating determinants) on health in later life and on outcomes related to socioeconomic status [mainly understood as employment (or educational level) and wages] The most efficient way (universal vs. group-specific interventions) to solve life cycle inequalities in health and socioeconomic status is an open question Alternative specifications should be used for the model, or long panels should be used to follow the same individuals over a period of time, as their age could help to understand the impact of health on socioeconomic status and to predict future health and the expenditure required

Furthermore, it would be interesting to take into account the existing links between parental socioeconomic status (measured by education, income or labor status) and child health and therefore between the health of a child today and his or her health and status in the future (its derived results in education, income and/or adult occupation) (Currie and Madrian 1999; Aizer and Currie 2014; Fletcher 2014; Flores and Kalwij 2014; Flores et al. 2015).

## Discussion

In this article we analyze the literature that studies the determinants of health with special attention to the relationship between socioeconomic status and health status. The socioeconomic status will be approached through different indicators, mainly income. To do so, we first discuss relevant articles in this field, which are among the most cited by literature, and then focus on a systematic literature review of recent years. In the revised literature, 5 of the 26 studies analyzed use aggregated data (19%) compared to 21 using individual data (81%). Most of them analyze the effect of income inequality on health status (17) in comparison with the 11 studies that consider income as a main variable of the study (2 of the studies consider both the absolute level of income and the distribution of income). The indicator of inequality most used in the literature is the Gini index.

The revised literature shows that people who live in areas of high inequalities tend to have a shorter life expectancy and high adult mortality and that this tendency increases over time. Among the studies that conduct their analysis on individual data, the results show a strong positive effect of income on health. The effect is particularly relevant in areas of high inequalities, and its influence can be observed from different socioeconomic measures (education, income, labor status).

Findings vary according to the type of study if the individual age is considered. In this sense, we find articles that show that individuals are statistically more likely to report poorer health if they were more unequally distributed during the first years of their lives than at an advanced age. However, other studies find that the magnitude of health inequalities is not consistent across age groups. For the income level, most of the results find that the major driver of the disequalizing effects of mortality is the positive association between old age and poverty.

There is also an interest in solving the apparent paradox that income appears to be related to health within countries but not between them. The explanation relies on the fact that in developed countries, which have already achieved a certain standard of living, increases in per capita GDP have little effect on the levels of health because of the *epidemiological transition* (understood under the fourth proposition by Omran (1971, 1982): “The shifts in health and disease patterns that characterize the epidemiologic transition are closely associated with the demographic and socioeconomic transition that constitute the modernization complex”), as in addition to epidemiological changes or changes in health conditions, the health transition also incorporates related social changes as a health care transition, as has been shown, for example, by Karlsson et al. (2010), Petrie et al. (2011), Hosseinpoor et al. (2012) and Siegel et al. (2014).

However, population health would also help to explain differences in income levels between individuals and between countries. The importance of investment in health has been re-emphasized by the theories of human capital. Improvements in health diminish productivity losses caused by disease in the workforce, reducing disability, weakness and the number of days off work. Also, they increase assistance to schools and the learning capacity of school children. One could also point to the decline of family disruption and other undesirable social issues as well as the reduction of negative externalities, for example, in the case of caring for the sick.

The effects of productivity gains in workers are particularly great for countries with a low level of development. Poor people have a higher risk of illness, and their income depends exclusively on their physical work. Investment in health would therefore be a productive investment, since it would increase income. It would be an important part of development and would help to reduce the income gap between rich and poor countries. Testing this relationship may lead to inconsistencies because of the causality between the two variables. This reverse causality could bias the results and make it difficult to draw inferences about the structural effect of income on health.

Finally, there are some limitations to this review we should consider. First, the literature search was limited to the main (three) databases. Future systematic reviews could also include other relevant sources. Second, the findings were not weighted for sample size.

## Conclusion

The published health economics literature on socioeconomic status, health and non-communicable diseases is characterized by many papers showing the complexity of those relationships. Improving this information is crucial if we are to capture the value of socioeconomic measures fully and to discover the most relevant determinants of health and non-communicable diseases.

From the literature analysis, we can conclude that income inequality was associated with worse average health. These results remain practically coincident regardless of the health indicator considered. The main conclusion of the studies analyzing the temporal evolution of both variables is that income inequalities in health increase over time to the detriment of the economically disadvantaged.

What is true is that different types of analysis produce very different results on the role of health determinants. Thus, the individual conception of health provides a different framework of research from a social analysis. The differences are relevant when the results are presented in terms of effectiveness in health policies and welfare (Wildman 2003). Although the determinants of health identified in individual studies are important variables in an aggregate analysis, there are specific factors that affect social groups. In this sense, for example, a better health status derived from a greater level of education may be the result of an education variable directly influencing the individual’s health or may be because of an improvement in social class due to a better education.

Finallly, further research is necessary to investigate the role of income level, its composition and its distribution in health status and the labor market. To help with this, perhaps we can highlight the greater potential of individual studies, with the new databases available, for analyzing hypotheses about a more detailed relationship among socioeconomic status, health and non-communicable diseases.

## Appendix

**Table 4:** Search strategy: PubMed, Cochrane Library and Web of Science

#	Search term
PubMed	
#1	Health [title/abstract]
#2	Income [title/abstract]
#3	Inequality [title/abstract]
#4	Limit to: journal article; year of publication ≥ 2010; English and Spanish; Humans subjects, free-full text
Cochrane Library	
#1	Health [title/abstract]
#2	Income [title/abstract]
#3	Inequality [title/abstract]
#4	Limit to: year of publication ≥ 2010.
Web of Science	
#1	Health [topic]; [title]
#2	Income [topic]; [title]
#3	Inequality [topic]; [title]
#4	Limit to: journal article; year of publication ≥ 2010; English and Spanish; Public Environmental Occupational Health “or” Social Issues “or” Health Care Sciences Services
